# Soil health indicators impacted by long-term cattle manure and inorganic fertilizer application in a corn-soybean rotation of South Dakota

**DOI:** 10.1038/s41598-019-48207-z

**Published:** 2019-08-13

**Authors:** Ekrem Ozlu, Saroop S. Sandhu, Sandeep Kumar, Francisco J. Arriaga

**Affiliations:** 1 Department of Soil Science, University of Wisconsin-Madison, Madison, WI 53706 USA; 2 Department of Agronomy, Horticulture and Plant Sciences, South Dakota State University, Brookings, SD 57007 USA; 3 Department of Soil and Water Sciences, University of Florida, Gainesville, Florida USA

**Keywords:** Environmental impact, Environmental health, Microbial ecology

## Abstract

Manure impacts labile pools of soil organic carbon (SOC) and nitrogen (N) which can influence soil microbial composition (MCC) and enzyme activities, and hence soil health. The present study was conducted to investigate the impacts of long-term dairy manure and inorganic fertilizers (INF) on soil carbon (C) as well as nitrogen (N) fractions, enzyme activities, and microbial community structure in different time horizons at planting (P), one month after planting (1MAP), and after harvesting (H) under corn (*Zea mays* L.)-soybean (*Glycine max* L.) rotation. Study treatments included three manure application rates (low, phosphorus-based recommended rate; medium, nitrogen-based recommended rate; and high, the double rate of medium nitrogen based recommended rate), two INF rates (medium only nitrogen additions; and high nitrogen, phosphorus, potassium, zinc, and sulfur additions) and a control (no application of manure and/or inorganic fertilizer). In comparison to the INF, the dairy manure not only significantly increased chemical fractions of C and N but also impacted the enzyme activities. Average urease activity after manure was applied was shown to be 26.8% higher than it was with INF applied at planting. The β-Glucosidase activity was 6 and 14% higher with manure than it was with INF at 1MAP and harvesting, respectively. The cold-water extractable nitrogen (CWEN) was enhanced with high manure rate at all timings of sampling compared to the high fertilizer rate (53%), and CK (90%). Principal component analysis indicated that MCC under manure differed from those under the INF treatments. The total bacteria/total fungi ratio at planting was increased with the INF compared to the manure addition. Pearson’s correlation analysis showed that CWEC, CWEN, and enzyme activities especially β-Glucosidase activity were the key determinants of MCC. Data from this study showed that, compared to inorganic fertilizers, manure can be beneficial in enhancing soil health indicators.

## Introduction

Soil microbial activity is an important component of soil health. It has been emphasized more recently that diversity in MCC is critical to maintain soil health^1,2^ due to their contributions to soil structure formation, decomposition of soil organic matter (SOM), and the biogeochemical cycling of nutrients^3^. Therefore, understanding the composition (distribution of soil microbes; PLFA) and activities (enzyme activities: urease and β-glucosidase activity) of soil microbes is increasingly recognized due to their importance to restore and sustain the ecosystems^4^. Application of manure, either alone or in combination with inorganic fertilizer (INF) increases soil organic carbon (SOC) concentration^5^ which appears to be more effective in maintaining or restoring soil organic matter (SOM) than the INF alone^6^.

SOM is often complex which is degraded by microbial enzyme activities^7^. The β-Glucosidase enzyme plays a major role in the degradation of SOM and plant residues due to its influences on catalyzing the hydrolysis of cellulose, the most abundant polysaccharide, for providing simple sugars for the soil microbial population^8^. Cattle manure is a valuable resource as a soil fertilizer because it provides high contents of macro- and micro-nutrients for crop growth and also a low-cost alternative to INFs^9^. Applications of dairy manure was reported to maintain or increase C and MCC^58^ in compare to chemical fertilization^57^. Similarly, some other studies found that dairy manure increased bacterial populations, substrate richness, FAME biomarkers for gram-negative organisms in all soils, and soil enzyme activities^59^. Soil enzyme activities are sensitive to soil management practices, such as applications of livestock manures, crop residues, municipal refuse, herbicide^10^, and tillage practices^11,12^.

Carbon (C) is the energy source for microbial tissues, whereas nitrogen (N) plays a critical role in crop production. Chemical C fractionations can be an option to better understand carbon stabilization^13–15^. Long-term application of manure increases not only the labile but also the recalcitrant pools of SOC, emphasizing the need for continued application of organic amendments for permanence of the accrued C^16^. In general, application of INF unable to enhance SOC^17^ and labile C fractions^18^ compare to manure additions. Changes in SOC content due to management practices and/or ongoing degradation or recovering processes can be visible in the labile fraction of soil C^19,20^. Chemical fractionations of soil C fuel the soil food web and greatly influence nutrient cycles and many biologically related soil properties^21^. Therefore, it is important to monitor the response of soil C fractions to soil management practices such as manure and INF application to soil.

Enzyme activities are correlated with the soil microbial communities, however; changes in microbial activities are just to reflect long-term changes in microbial diversity and not always the present microbial population^8^. The INF addition can be harmful for microorganisms^22^ owing to its negative impact on SOM and biological activity. Yet, short-term inorganic N applications had limited effects on soil enzyme activities and microbial biomass C^23^. Therefore, mechanisms behind enzyme activities, C and N fractions, microbial populations, and their associations are necessary to study. Phospholipid lipid fatty acids (PLFA) can be used to describe viable microbial communities in terms of total biomass and MCC^24^. Changes in the PLFA patterns related to key properties including C/N ratio, temperature, and bulk density those are the major drivers of the dynamics of the microbial community^25^. Manure applications typically results in increased soluble organic C in soil, therefore, its addition increases monounsaturated PLFA and supports higher levels of microbial activity compared to the INF^26^.

Besides SOC or N content, studies related to measuring the response of long-term application of manure and INF on enzyme activities and their relations to C fractions and microbial diversity are limited. Thus, the hypothesis of this study indicates that different application rates of manure and INF can influence C stability and hence improve the soil health by controlling C-N fractions, enzymes activities and MCC. Specific objective of the study was to assess the impact of manure and INF on soil enzyme activities (Urease activity and β-Glucosidase activity), carbon and nitrogen fractions, and MCC.

## Results

### Soil enzyme activity

**Figure 1:**
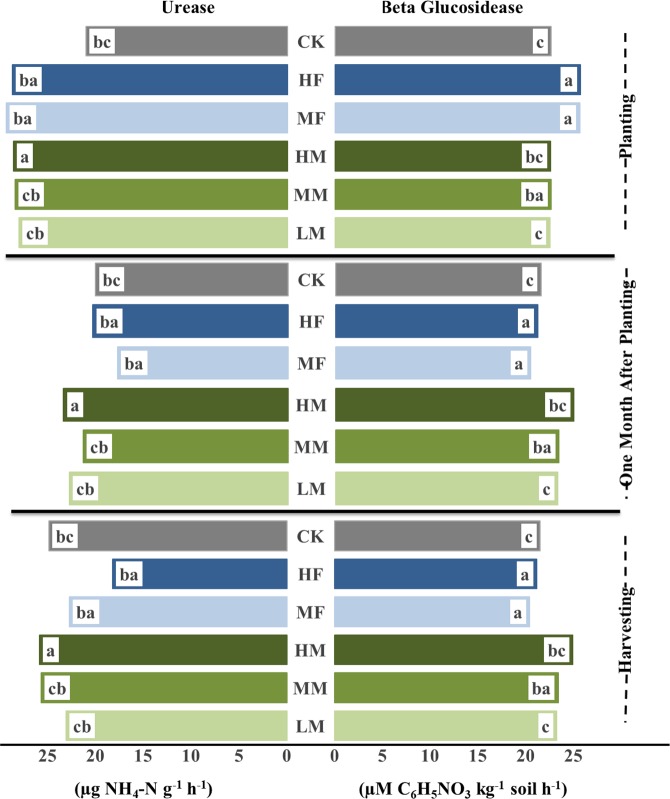
Soil enzyme activities as impacted by long-term manure and fertilizer treatments for 0–7.5 cm depth at soybean planting (P), one month after soybean planting (1MAP) and at soybean harvest (H). ^†^Mean values followed by different lower letters between each treatment within each sampling time represent significant differences due to manure and inorganic fertilizer application at *P* < 0.05. ^††^LM, low manure rate based on recommended phosphorus rate; MM, medium manure rate based on recommended nitrogen rate; HM, high manure rate based on double of the recommended nitrogen rate; MF, recommended fertilizer; HF, high fertilizer; and CK, control with no manure application.

The activity of enzymes increased with medium manure application relative to LM, HM, and INF. On an average, soil urease activity was significantly increased with the manure application in comparison to the INF rates and control treatment at planting and harvesting where INF decreased urease activity in comparison to control and manure addition at 1MAP (Fig. 1). Urease activity was also significantly different for contrast *M vs. F*. (p < 0.0001) where, on an average, M had 26.8% higher activity compared to the INF at planting. Similar trends were observed at 1MAP but difference was not significant at harvesting. For individual treatments, the CK treatment recorded the lowest value of soil urease activity at planting and harvesting. Urease activity under MM (28.5 µg NH_4_-N g^−1^ soil h^−1^) was significantly higher than those under MF (22.5 µg NH_4_-N g^−1^ soil h^−1^) at planting. Similarly, HM (19.7 µg NH_4_-N g^−1^ soil h^−1^; 27.3 µg NH_4_-N g^−1^ soil h^−1^) resulted significantly higher urease activity than those under HF (18.5 µg NH_4_-N g^−1^ soil h^−1^; 19.8 µg NH_4_-N g^−1^ soil h^−1^) at 1MAP and at harvesting.

Average soil β-Glucosidase enzyme activity under manure application was significantly greater in comparison to those under INF and control at 1MAP and harvesting (Fig. 1). Furthermore, β-Glucosidase was significantly different for contrast *M vs. F* (p < 0.03 at 1MAP and p < 0.01 at harvesting). The manure (M) had 6 and 14% higher activity compared to those under INF at 1MAP and harvesting, respectively. Moreover, β-Glucosidase activity under HM (24.8, and 27.8 µmol p-nitrophenol g^−1^soil h^−1^) were significantly greater compared to those under INF rates and control at planting and harvesting.

Soil urease activity was always greater in all the treatments at planting compare to 1MAP and harvesting, whereas, β-Glucosidase activity under manure addition was greater at harvesting compared to the other sampling times. Increasing rate of manure addition also increased the urease and β-Glucosidase activities at harvesting but observations at planting and 1MAP did not show similar trends (Fig. 1).

### Soil organic carbon fractions. generally, manure application increased the relative extractable hot and cold water C fractions, while impact on acid C fractions were not as marked

The highest values for CWEC and HWEC were monitored under HM at all sampling times, whereas, the lowest observations were monitored under CK except HWEC at planting (Table 1). The CWEC under HM at planting (0.41 g kg^−1^) was 2.73, 3.15, 3.42, 3.73, and 4.56 times higher than those under MM (0.15 g kg^−1^), LM (0.13 g kg^−1^), HF (0.12 g kg^−1^), MF (0.11 g kg^−1^), and CK (0.09 g kg^−1^), respectively. Similar trends were observed at 1MAP and at harvesting for both the CWEC and HWEC. In addition, *M vs. F* contrast was also significant in terms of CWEC and HWEC at all sampling times except for HWEC at 1MAP. The HWEC under HM at planting (1.02 g kg^−1^) was the highest and MF (0.5 g kg^−1^) the lowest. The average AEC was significantly greater under manure application in comparison to the control and INF rates at planting and harvesting, but differences were not always significant. The AEC values were higher with the application of HM at planting and MM at harvesting, whereas, lower values were observed under HF at planting and under MF at harvesting. Overall, HM increased C fractions in comparison to the other manure rates, INF rates, and control, whereas, INF either decreased or did not impact C fractions compare to control.

**Table 1:** Soil carbon fractions (CWEC, cold water extractable carbon; HWEC, hot water extractable carbon; AEC, acid extractable carbon) as impacted by long-term manure and fertilizer treatments for 0–7.5 cm depth at soybean planting (P), one month after soybean planting (1MAP) and at soybean harvest.

Treatments	CWEC			HWEC			AEC		
	*------------------------------------------(g kg* ^*−*1^ *) --------------------------------------------*								
	P	1MAP	H	P	1MAP	H	P	1MAP	H
LM^††^	0.13^b†^	0.19^b^	0.09^dc^	0.58^b^	0.89^b^	0.78^ba^	8.50^a^	9.87^ns^	8.99^b^
MM	0.15^b^	0.17^cb^	0.14^ba^	0.62^b^	0.66^cb^	0.84^ba^	9.81^a^	8.55	11.4^a^
HM	0.41^a^	0.34^a^	0.17^a^	1.02^a^	1.37^a^	1.04^a^	10.2^a^	8.43	11.0^a^
MF	0.11^b^	0.09^d^	0.07^d^	0.50^b^	0.67^cb^	0.65^cb^	8.20^a^	7.19	7.48^c^
HF	0.12^b^	0.13^dc^	0.12^cb^	0.55^b^	0.73^cb^	0.66^cb^	5.82^b^	8.61	8.73^cb^
CK	0.09^b^	0.08^d^	0.07^d^	0.53^b^	0.47^c^	0.56^c^	8.10^a^	7.17	7.61^cb^
	**Analysis of Variance (*P*** > ***F*)**								
*Treatment*	0.001	<0.0001	0.0001	0.0001	0.005	0.011	0.013	0.178	0.0002
M vs. F	0.019	<0.0001	0.007	0.004	0.058	0.006	0.010	0.029	0.003

^†^Mean values followed by different lower letters between each treatment within each sampling time represent significant differences due to manure and inorganic fertilizer application at *P* < 0.05. ^††^LM, low manure rate based on recommended phosphorus rate; MM, medium manure rate based on recommended nitrogen rate; HM, high manure rate based on double of the recommended nitrogen rate; MF, recommended fertilizer; HF, high fertilizer; and CK, control with no manure application. ns, no significant.

### Soil nitrogen fractions

The higher values for nitrogen fractions (CWEN, HWEN, and AEN) were monitored under HM manure application rate, whereas, lower values were observed under CK except that HF resulted lowest AEN at planting. Overall, manure addition increased N content in the form of chemically extractable fractions compared to the INF and CK. However, this statement cannot be concluded for lower rates of individual manure applications (MM and LM). The CWEN ranged from 0.137 g kg^−1^ under HM at 1MAP to 0.015 g kg^−1^ under CK at harvesting (Table 2). Similar trends were monitored in terms of HWEN at 1MAP and harvesting, and AEN at 1MAP. Moreover, *M vs. F* contrast was also significant in terms of CWEN and HWEN at all sampling times and in terms of AEN at harvesting.

**Table 2:** Soil nitrogen fractions (CWEN, cold water extractable nitrogen; HWEN, hot water extractable nitrogen; AEN, acid extractable nitrogen) as impacted by long-term manure and fertilizer treatments for 0–7.5 cm depth at soybean planting (P), one month after soybean planting (1MAP) and at soybean harvest.

Treatments	CWEN			HWEN			AEN		
	*------------------------------------------(g kg* ^*-1*^ *) --------------------------------------------*								
	P	1MAP	H	P	1MAP	H	P	1MAP	H
LM^††^	0.039^b†^	0.043^b^	0.024^b^	0.100^cb^	0.147^b^	0.126^cb^	1.632^ac^	1.994^ns^	1.567^b^
MM	0.045^b^	0.032^b^	0.041^a^	0.116^b^	0.114^cb^	0.142^b^	1.862^ba^	1.669	2.134^a^
HM	0.091^a^	0.137^a^	0.048^a^	0.206^a^	0.190^a^	0.187^a^	2.060^a^	1.598	2.165^a^
MF	0.037^b^	0.034^b^	0.020^b^	0.072^d^	0.074^ed^	0.094^cd^	1.747^ba^	1.519	1.239^b^
HF	0.041^b^	0.043^b^	0.017^b^	0.079^cd^	0.107^cd^	0.087^d^	1.179^c^	1.716	1.522^b^
CK	0.029^b^	0.035^b^	0.015^b^	0.075^cd^	0.064^e^	0.078^d^	1.473^bc^	1.382	1.483^b^
	**Analysis of Variance (*P*** > ***F*)**								
*Treatment*	0.003	0.001	0.003	<0.0001	<0.0001	0.0001	0.0224	0.1396	0.0023
M vs. F	0.034	0.016	0.002	<0.0001	0.0006	0.0006	0.0694	0.2505	0.0003

^†^Mean values followed by different lower letters between each treatment within each sampling time represent significant differences due to manure and inorganic fertilizer application at *P* < 0.05. ^††^LM, low manure rate based on recommended phosphorus rate; MM, medium manure rate based on recommended nitrogen rate; HM, high manure rate based on double of the recommended nitrogen rate; MF, recommended fertilizer; HF, high fertilizer; and CK, control with no manure application. ns, no significant.

### Soil microbial community structure

Total PLFA biomass was not sensitive in detecting significant differences between treatments, however, there were significant differences in total bacteria, total fungi, and their ratio. Under all treatments, total abundance of gram positive (G+) bacteria, gram negative (G−) bacteria and actinomycetes at planting and 1MAP were considerably higher than the other MCC (Table 3). The arbuscular mycorrhiza fungi (AMF) increased under HM (4.15% mol) than those under MM (4.01% mol), CK (3.60% mol), HF (3.49% mol), MF (3.39% mol), and LM (3.21% mol) by 3.5%, 15.3%, 18.9%, 22.4%, and 29.3% at the planting, respectively. This indicates that manure increased the AMF in comparison to the same N rate of INF and control in spring. The trend for AMF at 1MAP was similar to the trend observed at planting where application of manure addition increased the average AMF by 10.2% at planting and 28.1% at 1MAP in comparison to those under INF addition with significant contrasts for *M vs. F* (p < 0.02 at planting), respectively. Average actinomycetes were decreased with manure in comparison to the INF at both planting and 1MAP (Table 3). Similarly, contrasts for *M vs. F* at planting (p < 0.01) and 1MAP (p < 0.009) were statistically significant. Differences in G−, G+, anaerobe, fungi, and eukaryote were not significant at planting, whereas, G- was significantly higher under CK compared to manure application rates, whereas, the lowest values were observed under INF at 1MAP. Moreover, eukaryote was significantly increased with INF than the manure and control only at 1MAP. In addition, contrast for *M vs. F* were significant in terms of eukaryote (p < 0.03).

**Table 3:** Soil microbial community composition as impacted by long-term manure and fertilizer treatments for 0–7.5 cm depth at soybean planting (P).

Treatments	G−		G+		Anaerobe		Actinom		AMF		Fungi		Eukaryote	
	P	1MAP	P	1MAP	P	1MAP	P	1MAP	P	1MAP	P	1MAP	P	1MAP
	*------------------------% ------------------------*													
LM^††^	45.55^ns^	47.91^b^	15.38^ns^	22.30^ns^	1.79^b^	2.85^ns^	16.86^ba^	11.86^b^	3.21^c^	3.36^bc^	5.31^ns^	4.18^ns^	11.91^ns^	6.44^b^
MM	45.35	50.54^b^	16.34	19.65	3.44^a^	1.66	15.97^bc^	12.54^ba^	4.01^ba^	5.00^ba^	5.51	6.29	9.39	4.34^b^
HM	45.73	51.36^b^	17.44	19.13	3.12^ba^	2.04	13.67^c^	12.77^ba^	4.15^a^	5.72^a^	4.93	5.41	10.97	3.59^b^
MF	42.68	44.77^b^	17.61	16.86	3.92^a^	1.23	18.18^ba^	16.25^a^	3.39^c^	3.41^bac^	5.38	4.46	8.84	13.04^a^
HF	41.32	48.63^b^	23.08	18.98	3.89^a^	1.52	18.31^ba^	15.65^ba^	3.49^bc^	3.92^bac^	2.72	4.92	7.20	6.38^b^
CK	45.46	70.85^a^	15.54	9.80	3.36^a^	1.94	18.84^a^	7.73^c^	3.60^bac^	1.70^c^	5.31	2.89	7.89	5.09^b^
	**Analysis of Variance (P** > **F)**													
Treatment	0.9	0.001	0.06	0.07	0.05	0.3	0.009	0.006	0.04	0.04	0.2	0.07	0.3	0.004
M vs. F	0.3	0.3	0.06	0.4	0.06	0.07	0.01	0.009	0.02	0.2	0.3	0.4	0.09	0.03

^†^Mean values followed by different lower letters between each treatment within each sampling time represent significant differences due to manure and inorganic fertilizer application at *P* < 0.05. ^††^LM, low manure rate based on recommended phosphorus rate; MM, medium manure rate based on recommended nitrogen rate; HM, high manure rate based on double of the recommended nitrogen rate; MF, recommended fertilizer; HF, high fertilizer; and CK, control with no manure application. ns, no significant.

**Figure 2:**
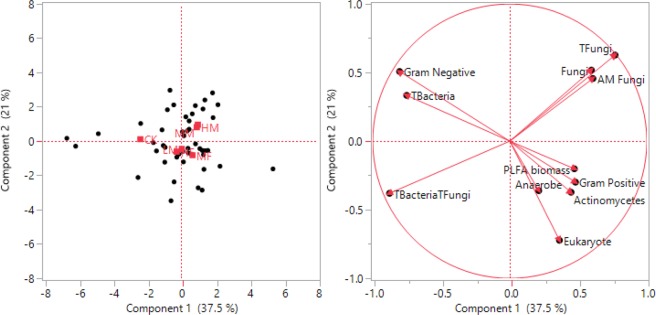
Principal components analysis of phospholipid fatty acid (PLFA) profiles as impacted by long-term manure and fertilizer treatments for 0–7.5 cm depth at soybean planting (P), one month after soybean planting (1MAP) and at soybean harvest.

The principal components analysis determined differences in microbial community structure under manure and INF at two different sampling times (planting and 1MAP). The first principal component (PC1) accounted for 37.5% of the total variation, and the second component (PC2) for 21.0% of the variation (Fig. 2). This demonstrates the difference in MCC under six treatments at two different sampling times. There was a high degree of variability with a strong separation based on different sampling times. Differences for G+/G− bacteria ratio and total fungi at planting, and total PLFA biomass at 1MAP were not significant at planting (Table 4). Overall, manure application increased G+/G− bacteria ratio and total fungi compare to INF and control at 1MAP. In contrast, Bacteria/Fungi ratio (B/F) was significantly higher under INF in comparison to manure addition at both planting and 1MAP (Table 3). However, total bacteria were significantly higher under CK compared to HM (5.9%), LM (6.4%), HF (6.5%), MM (7.1%), and MF (14.2%). This suggests that overall manure addition (84.86% mol) increases the total bacteria 1MAP in comparison to INF (81.94% mol) with p < 0.0001. A similar trend was observed in terms of total fungi at 1MAP with p < 0.001. In addition contrast of *M vs. F* for total bacteria (p < 0.047) was significant at 1MAP. This statement indicates that not only organic or inorganic fertilizer additions but also rate of both manure and INF additions resulted significantly different values for these properties for both planting and 1MAP.

**Table 4:** Soil PLFA community composition as impacted by long-term manure and fertilizer treatments for 0–7.5 cm depth at soybean planting (P) and one month after planting (1MAP).

Treatments	G+/G− Bacteria		Total Bacteria		Total Fungi		Bacteria/Fungi Ratio		Total PLFA Biomass	
	P	1MAP	P	1MAP	P	1MAP	P	1MAP	P	1MAP
			*------------------------% mol------------------------*						*-nmol gr* ^*-1*^ *soil-*	
LM^††^	0.34^ns†^	0.47^ns^	79.58^ns^	84.91^b^	8.51^ns^	7.53^b^	9.42^b^	12.74^b^	79.91^ns^	87.07^ns^
MM	0.36	0.39	81.09	84.37^b^	9.53	11.29^a^	9.09^b^	7.48^b^	104.2	78.21
HM	0.38	0.37	79.96	85.29^b^	9.07	11.12^a^	8.81^b^	7.83^b^	117.9	94.75
MF	0.41	0.38	82.39	79.10^c^	8.77	7.87^b^	9.42^b^	10.34^b^	90.58	56.26
HF	0.56	0.39	86.6	84.79^b^	6.20	8.84^ba^	14.41^a^	9.74^b^	82.47	85.52
CK	0.34	0.14	83.21	90.32^a^	8.91	4.59^c^	10.17^b^	21.51^a^	27.15	57.53
	**Analysis of Variance (P > F)**									
Treatment	0.3	0.09	0.2	0.0001	0.2	0.001	0.04	0.0006	0.5	0.3
M vs. F	0.1	0.9	0.05	0.047	0.2	0.1	0.08	0.8	0.4	0.4

^†^Mean values followed by different lower letters between each treatment within each sampling time represent significant differences due to manure and inorganic fertilizer application at *P* < 0.05. ^††^LM, low manure rate based on recommended phosphorus rate; MM, medium manure rate based on recommended nitrogen rate; HM, high manure rate based on double of the recommended nitrogen rate; MF, recommended fertilizer; HF, high fertilizer; and CK, control with no manure application. ns, no significant.

## Discussion

While addressing the global warming outcomes, one should consider soil enzyme activities that may affect recalcitrant humid matter^27^ owing to their association with changes in temperature^8^. The balance between these two competing processes determines how much C is sequestered and stabilized as well as contributing to microbial diversity and activity, and a host of enzyme properties that determine soil fertility and plant productivity^28^. Soil enzymes have significant association with temperature, moisture^29,30^, soil porosity, and GHG emissions^31^.

In this study, urease and β-Glucosidase activities were higher in average with manure addition compared with those under INF and control in all the soil sampling times examined. Long-term addition of INF can change the amount of enzymes released by microbes. In the previous studies, it has been documented that SOC and N contents increased with the addition of manure compared to INF^17^. Similarly, water stable aggregates found to be higher with the addition of manure compared to INF^17^. In comparison to manure addition, the application of INF addition decreased the enzyme activities by limiting their energy and production by (i) decreasing the SOC, which is the energy source for soil microbial diversity and maintenance of their structure and functional capacity, (ii) declining the soil N content, which is the production source, and (iii) breaking down the soil aggregates. It is well known that soil physical and chemical environments are related to fertility and crop management systems, and are major determinants of microbial populations and activities^32^. Manure increases SOM, soil aggregation and N content, decreases soil bulk density and maintain the soil pH^17^ those are beneficial in supporting soil microbial activities. The β-Glucosidase activity, which usually increased with the increase in soil microbial biomass, would reflect on a soil’s ability to break down plant residues and improve the availability of nutrients for subsequent crops^8^. More optimal soil water content combined with greater levels of organic C and N, nutrient cycling, and a large amount of N is stored in the relatively labile microbial biomass which is associated with greater microbial activity in manure amended soils^33^. Therefore, it is important to consider soil enzyme activities among the soil health indicators to provide a better understanding of microbial activities and C-N dynamics.

The SOC plays a critical role for better soil quality and higher crop productivity by influencing soil chemical, physical, and biological properties^34^. Soil management practices influence labile SOC pools which is moderately or easily decomposable, with higher turnover rate compared with recalcitrant fractions^35^. However, responses of labile SOC pools to manure additions are comparatively limited^16^. The results of the present study showed that, in general, manure significantly increased CWEC and HWEC compared to the INF and CK. Long-term manure applications are beneficial in order to sequester labile and recalcitrant pools of SOC^16^. In addition, increasing rate of manure addition increased CWEC and HWEC in comparison to lower rates of manure additions, however, INF did not show similar trend. A study by^36^ reported that INF (rates are 200 and 400 kg N ha^−1^ yr^−1^) decreased HWEC and soil microbial dynamics after 5 years. The lability of SOC refers to the comparatively easy to decomposable and available for microbes, whereas, recalcitrant C is structurally complex or physically protected by soil minerals^35^. However, there were no significant difference in terms of AEC at planting and 1MAP. Results of Šimon^18^ are also matching with our findings in terms of AEC, and AEN. Nevertheless, the C/N ratio was significantly lower for INF treatments early in the season and increasing over time, but differences were not significant to manure treatments at harvest (Table S4). These results highlight the complex connection between C and N cycles in soil. It has been well documented that impacts of OM additions such as manure on maintaining or restoring OM is higher in comparison to those under INF^6^.

The manure addition significantly enhanced CWEN, HWEN, and AEN compared to the INF and CK treatments at all sampling times. However, there were no significant difference in terms of AEN at planting and 1MAP. Besides the quantity, the quality of organic C and N is also important. The quality of organic C and N in agricultural lands are still slightly unknown. However, it is well documented that agricultural fields contribute higher water extractable OM in comparison to freshwater ecosystems^37^. Besides soil moisture and temperature, manure can highly influence soil water extractable OM. Ghani, *et al*.^36^ reported strong correlations between hot water extractable fraction, which could be used as an integrated measure of soil quality, with other biochemical properties. In this study, HWEC significantly correlated with β-Glucosidase activity (p < 0.015), total fungi (p < 0.035), and AMF (p < 0.007). It is proposed that decrease in HWEC would also refer the lowering in other labile organic nutrient pools such as N, because when HWEC is extracted, other labile organic nutrient pools are also extracted along with C^36^.

**Figure 3:**
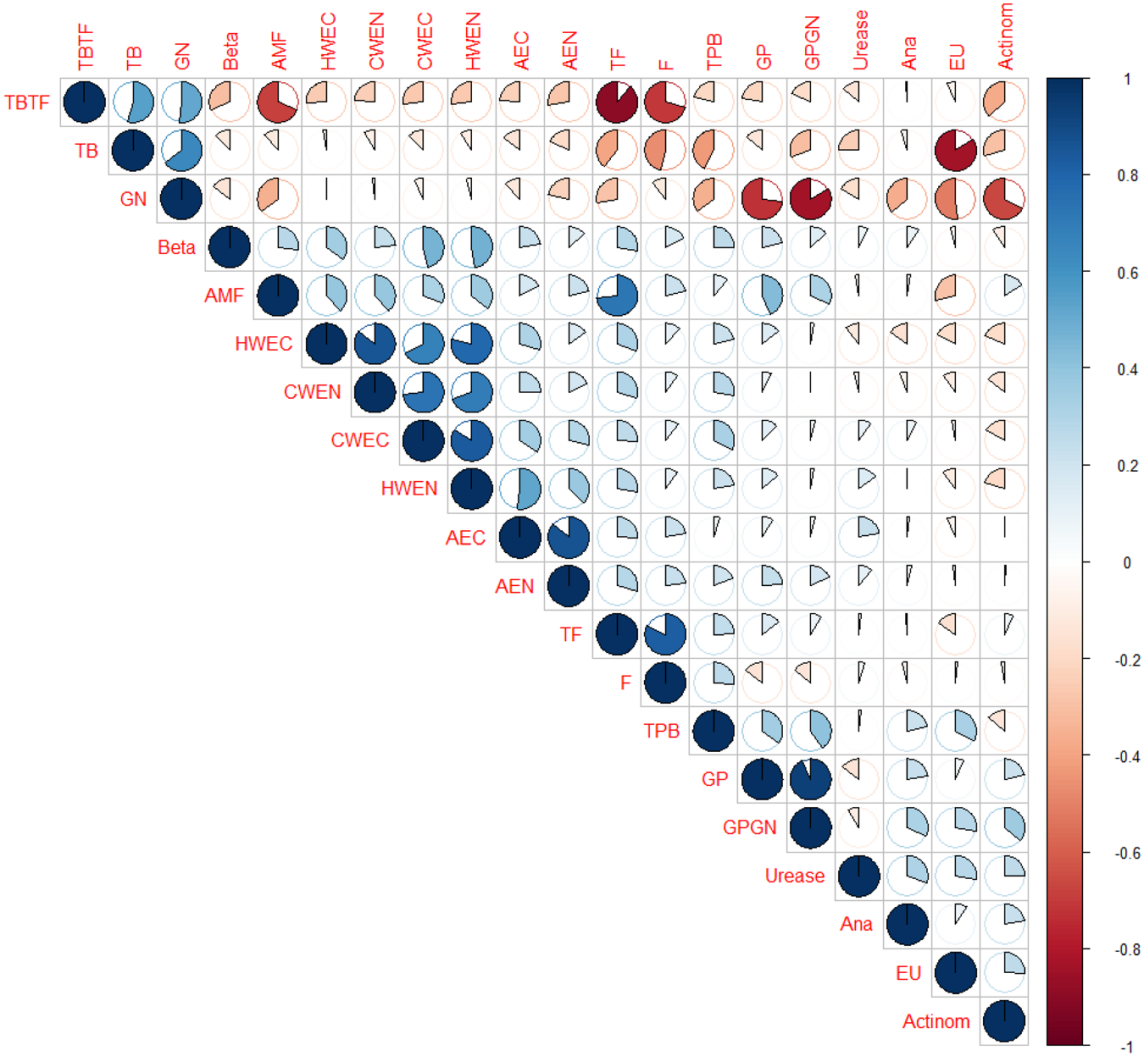
Pearson’s correlation analysis of microbial parameters and soil physicochemical properties as impacted by long-term manure and fertilizer treatments for 0–7.5 cm depth at soybean planting (P), one month after soybean planting (1MAP) and at soybean harvest. *p < 0.05; **p < 0.01; ***p < 0.001. The color and size of the pie chart denotes the magnitude and direction of the relationship. Actinom, actinomycetes; AEC, acid extractable carbon; AEN, acid extractable Nitrogen; AMF, arbuscular mycorrhiza fungi; Ana, anaerobes; Beta, beta-glucosidase activity; CWEC, cold water extractable carbon; CWEN, cold water extractable nitrogen; EU, eukaryote; F, fungi; GN, gram negative bacteria; GP, gram positive; GPGN, gram positive gram negative ratio; HWEC, hot water extractable carbon; HWEN, hot water extractable nitrogen; TB, total bacteria; TBTF, total bacteria and fungi ratio; TF, total fungi; TPB, total PLFA biomass; Urease, urease activity.

The correlation analysis revealed that enzyme activity was also significantly correlated with soil microbial parameters as estimated by the PLFAs. This was also supported by^38^, who reported a strong association between enzyme activities and microbial biomass^39^. Soil microbial communities are important for biogeochemical cycling due to their roles in decomposition. The percentage of G− bacteria was higher than the percentage of G+. The G− bacteria specially uses fresh plant inputs as C sources, whereas G+ bacteria use older or processed^40^. G+ bacteria were correlated with AEN and β-Glucosidase activity (Fig. 3), however, G− bacteria was negatively correlated with AMF, TF, total PLFA biomass, and urease activity. Moreover, β-Glucosidase activity was also positively correlated with HWEC, CWEC, HWEN, AMF, and total fungi, whereas, it was negatively correlated with TBTF ratio. Moreover, fungi amounts were correlated with AEC (P < 0.002), and AEN (P < 0.03). Furthermore, urease activity was positively correlated with anaerobes, eukaryotes, actinomycetes, AEC, and HWEN, whereas, negatively correlated with total bacteria. Thus, it can be reported that fungi and β-Glucosidase activity quickly respond to SOC inputs. This statement was also reported by^41^ in paddy soils. Compared to the INF, manure addition had significantly lower G+/G− ratio, and G− bacteria at 1MAP were observed. This might be owing to that the decrease in fresh organic substance (such as manure) movements increased G− bacterial and fungal development^39^. Further, the F/B ratio was significantly lower under INF addition than the manure were observed. A higher ratio of bacteria/fungi assigns environment friendly and sustainable agroecosystem management^42^.

There were significantly more actinomycetes at 1MAP under INF addition than under manure addition. Similar findings were also supported by^43^. In this study, we did not find any significance correlation of actinomycetes with other properties. This might be due to diversity in crop species, soil health and management, and their complex interactions^44^. The organic manure and INF additions can also indirectly impact microbial properties by enhancing critical soil nutrients such as N and phosphorus^45^. Since the addition of organic substances enhance the PLFA biomarkers for bacteria and fungi but reduce that for actinomycetes, whereas, INF shows opposite trend due to providing SOC and other nutrients deficiency, manure rises the bacterial and fungal communities higher than actinomycetes in comparison to INF additions^41^. Even though actinomycetes and G+ bacteria are the indigenous microbiota of soybean soil, the addition of organic manures provides a dominant representation of G− bacteria and fungi^41^. However, manure provide the high contents of easily degradable organic mixtures, whereas, the heterogeneity in the soil-manure composition might impact the additional biological substrates such as ammonia^46^.

The principal components analysis and Pearson’s correlation analysis revealed that C and N fractions are significant due to their correlation with MCC and enzyme activities. Microbial dynamics and soil physical structure are related. For instance, soil bulk density helps to enhance the interface between soil and water/air potential and hence biological and chemical exchange^16^. Bulk density and WAS were significantly positively correlated with addition of manure^17^ in the same field of this study. It was recently reported that other soil properties influence spatial distribution of soil microbes along with climate, topography and land use^55^. Moreover, it is known that INF addition to soil decline in soil pH and SOC^17^. Our findings are also supported by^47^, who stated that the AMF lowers with decline in soil pH and TOC. Hackl, *et al*.^47^ also mentioned that cis-11-palmitoleic acid enhanced the microbial properties and hence rises microbial biomass with high C contents in paddy soils. A high quantity of SOM provides the development in a large, active, microbial biomass which can decompose more fresh available composites^48^. Gram-positive bacteria, actinomycetes, fungi, and arbuscular mycorrhizal fungi were recently found to be significantly correlated with variability of soil carbon and nitrogen^56^. Urease were also very highly correlated with anaerobe (P < 0.02) whereas β-Glucosidase activity was highly correlated with Fungi (P < 0.006).

## Conclusion

In general, compared with the INF, manure addition increased urease and β-Glucosidase activities, and C-N fractions. Moreover, higher rate of manure addition also increased these properties but this was not observed with INF. CWEC and HWEC at all sampling times (planting, 1MAP, and harvesting) and AEC at both planting and harvest were increased by addition of manure compare to INF and control. Similar results were also observed in terms of N fractions (CWEN, HWEN, and AEN). The AMF was higher under manure additions compared to INF at both planting and 1MAP, whereas, INF increased anaerobes, actinomycetes, and eukaryotes than those under manure addition. Pearson’s correlation analysis showed that SOC and N fractions especially CWEC, CWEN, and enzyme activities were the key determinants of MCC. Overall, the manure addition strongly influenced microbial dynamics and structure, hence faster subsurface soil nutrient cycling. However, INF was also an important influence on some MCC such as actinomycetes. Therefore, we can conclude that manure addition influences the formation, storage, and turnover of SOC and N, and enzyme dynamics, as well as soil microecology.

## Materials and Methods

### Study area and experimental design

The study was conducted on a well-drained and nearly flat (slope <1%) silt loam Vienna soils (Fine-loamy, mixed, frigid Udic Haploborolls) under corn (*Zea mays* L.) and soybean (*Glycine max* L.) rotation at Brookings county of South Dakota, USA (44°22′07.15″N, 96°47′26.45″W) for 8-years (2008–2015). In general, this region has a humid continental climate having relatively humid summers and cold, and snowy winters with average annual temperature of −15.8 °C in the winter and 27.8 °C in the summer, and average annual rainfall of 637.15 mm, respectively. The present long-term study treatments were established in 2008 using a randomized complete block design with four replications and the plot size was 6 m (Width) by 18 m (Length). The study treatments included three manure application rates (low manure rate (LM), medium manure rate (MM), and high manure rate (HM)), two fertilizer application rates (medium INF rate (MF) and (ii) high INF rate (HF)), and a control (CK; neither organic manure nor mineral fertilizer N was applied). The LM rate is calculated according to phosphorus concentrations which considers P-based recommended rate of manure, whereas, MM is calculated according to the nitrogen content considering N-based recommended rate of manure according to Department of Environment and Natural Recourses (DENR) tool standards. The HM rate is doubled the amount of MM. The DENR tool calculate manure addition rates according to manure and soil phosphorus and nitrogen contents, and crop phosphorus and nitrogen requirements for a particular yield goal. The yield goal was 4.04 Mg ha^−1^ used for soybean and 9.42 Mg ha^−1^ for corn grain. Only, urea fertilizer was applied for MF, whereas, a combination of urea, MAP, potash, zinc-sulfate, and ammonium-sulfate have been applied for HF. Annual manure applications were established in spring just before planting. Further detail about the site can be found elsewhere^49^.

### Soil sampling and storage

Soil samples for 0–7.5 cm depth were collected three times in 2015 during soybean growing season; before planting in May, one month after planting (1MAP) in June, and after soybean harvest in late October. Four soil samples were collected from each plot, mixed to make a composite sample, immediately sealed in plastic bags, and moved to ice cooler for transporting to the lab and stored at −20 °C in freezer for less than a week to pursue pending analysis.

### Soil enzyme activity analysis

Urease activity was assayed according to the methods described by Kandeler and Gerber^50^. Briefly, 5 g of soil was placed into each of the three 50 ml incubation flasks. Two of them were treated with 2.5 ml of urea solution (720 mM) and 20 ml of borate buffer (0.1 M, pH 10), and third one was considered as control and only 20 ml borate buffer were added to it, and flasks were incubated for 2 h at 37 °C. After incubation, 2.5 ml of urea solution was added to control and 30 ml of potassium chloride (2 M) - hydrochloric acid (0.01 M) solution to all three flasks and shook for 30 min. All three soil suspensions were filtered using Whatman filter no. 4. After filtering the soil suspension, 5 ml of sodium salicylate-sodium hydroxide solution and 2 ml of sodium dichloroisocyanurate solution (3.91 mM) were added for color development. The absorbance of the color was measured at 660 nm using spectrophotometer. Also, to prepare standard curve, standards of 0, 1, 1.5, 2, and 2.5 µmol N ml^−1^ of ammonium chloride were prepared.

The β-Glucosidase enzyme activity was determined by placing 1 g of soil in three 50 ml Erlenmeyer flasks and 0.2 ml toluene was added in all the flasks, mixed and led it set for 15 minutes^51^. Then 4 ml of modified universal buffer (MUB, pH 6) was added to all the flasks and 1 ml of 50 mM p-nitrophenyl-β-D-glucoside (PNG) solution were added to only two flasks and third was considered as control. All three flasks were incubated for 1 h. After incubation, 1 ml of 0.5 M CaCl_2_ and 4 ml of 0.1 M THAM buffer (pH 12) were added to all three flasks, and 1 ml PNG solution was added to the control flask. Soil suspensions were filtered using Whatman No. 2 V folded filter paper. Yellow color intensity of the filtered solutions was measured with spectrophotometer at 405 nm. A calibration curve was developed with standards containing 0, 100, 200, 300, 400, and 500 nmol of p-nitrophenol solution in each flask.

### Chemical fractionation of soil organic carbon and nitrogen

Soil C and N fractions were analyzed using cold water, hot water and acid (1 Molar HCL) extraction methods^36,52^. Briefly 3 g soil was taken into 50 ml polypropylene centrifuge tubes, and 30 ml distilled water was added in each tube. Soil was mixed thoroughly with water on vertex mixer for 10 sec and then moved to end-over-end shaker for 30 minutes at 40 rpm. After shaking, the suspension was centrifuged at 3000 rpm for 25 minutes, and supernatant was separated from soil by using 0.45 µm pore size syringe filters and termed as cold-water extractable C and N (CWEC and CWEN). Further, 30 ml distilled water was added in each tube to determine hot water fraction and proceed same as cold water fraction. However, for hot water fraction, hot water bath (80 °C) was used to shake samples for 12–15 hours. Further, acid hydrolysis was carried out on the soil left after hot water extract by adding 15 ml of 1 M HCL to two different tubes to determine one molar fraction (AEC) at 105 °C for six hours. After the hydrolysis process, tubes were proceeded the same way as of CWEC Total C and N in all three extracts (CWEC, HWEC, and AEC) were determined using TOC-L analyzer (Shimadzu Corporation, model- TNM-L-ROHS). These total C and N were considered as organic C and N in each extract by considering no inorganic C in soil.

### Soil microbial community structure analysis

Total of Fifty eighth phospholipid fatty acids (PLFA) for each sample were determined and expressed in % mol and nmol·g^−1^ dry soil by the lipid extraction method^53^. Briefly, 3 g of soil was extracted in a one-phase mixture consisting of chloroform, methanol, and citrate buffer (1:2:0.8, vol vol^−1^ vol^−1^). After splitting the extracts into two phases by adding chloroform and buffer, the lipid-containing phase was dried under a stream of N and stored at −20 °C. The lipid material was fractionated on columns containing silicic acid into neutral and glyco- and phospholipid-containing polar lipids. Then, phospholipid fraction was dried under a stream of N and saved for preparations of fatty acid methyl esters. The phospholipids were subjected to a mild alkaline methanolysis, and the resulting fatty acid methyl esters were separated on gas chromatograph (GC) equipped with a flame ionization detector. Relative retention times of supposed fatty acid methyl esters were compared with those of standards. methyl nonadecanoate (19:0) were added at the beginning of extraction process. The total PLFA was calculated with 19:0 as the internal standard.

The concentration of PLFA from GC output was converted to nmol/g using the following equation

$${\rm{C}}[{\rm{nmol}}/{\rm{g}}]=\frac{Ffm\ast Cis\ast 2\ast 1000\ast 100}{EW\ast TS\ast Fis\ast MG}$$

Where C is the concentration of the PLFA in nmol/g, Ffm is the area of the phospholipid methylester, Cis is the concentration of the internal standard, EW is the weight of soil in grams, TS is the dry matter of soil in % (100/TS = dry matter factor), Fis is the area of internal standard, and MG is the molecular weight of the phospholipid methylester in ug/umol, 2 is the variable factor and 1000 is the factor to obtain nmol.

### Statistical analysis

The one-way ANOVA was used to determine differences in individual treatments whereas paired t-test were placed to examine differences in overall manure with overall INF at *α* < 0.05^54^. In addition, Pearson’s correlation analysis was conducted to investigate relationships between soil C-N fractions and enzyme activities, and microbial parameters by using JMP package of SAS 9.3^54^. Differences in MCC were investigated using principal components analysis in JMP package of SAS 9.3^54^.

## Supplementary information

Supplementary Info

## References

[1] Mele PM, Crowley DE (2008). Application of self-organizing maps for assessing soil biological quality. Agriculture, Ecosystems & Environment.

[2] Shen W (2008). Land use intensification affects soil microbial populations, functional diversity and related suppressiveness of cucumber Fusarium wilt in China’s Yangtze River Delta. Plant Soil.

[3] PAUL E.A. (2007). SOIL MICROBIOLOGY, ECOLOGY, AND BIOCHEMISTRY IN PERSPECTIVE. Soil Microbiology, Ecology and Biochemistry.

[4] Potthoff M (2006). Soil microbial community composition as affected by restoration practices in California grassland. Soil Biology and Biochemistry.

[5] Gong W, Yan X-y, Wang J-y, Hu T-x, Gong Y-B (2009). Long-term manuring and fertilization effects on soil organic carbon pools under a wheat–maize cropping system in North China Plain. Plant Soil.

[6] Wu T (2004). Influence of cultivation and fertilization on total organic carbon and carbon fractions in soils from the Loess Plateau of China. Soil and Tillage Research.

[7] Burns RG (2013). Soil enzymes in a changing environment: current knowledge and future directions. Soil Biology and Biochemistry.

[8] Stott D, Andrews S, Liebig M, Wienhold BJ, Karlen D (2010). Evaluation of β-glucosidase activity as a soil quality indicator for the soil management assessment framework. Soil Science Society of America Journal.

[9] Lazcano C, Gómez-Brandón M, Domínguez J (2008). Comparison of the effectiveness of composting and vermicomposting for the biological stabilization of cattle manure. Chemosphere.

[10] García-Ruiz R, Ochoa V, Hinojosa MB, Carreira JA (2008). Suitability of enzyme activities for the monitoring of soil quality improvement in organic agricultural systems. Soil Biology and Biochemistry.

[11] Mijangos I, Pérez R, Albizu I, Garbisu C (2006). Effects of fertilization and tillage on soil biological parameters. Enzyme and Microbial Technology.

[12] Ekenler M, Tabatabai M (2003). Effects of liming and tillage systems on microbial biomass and glycosidases in soils. Biology and Fertility of Soils.

[13] von Lützow M (2007). SOM fractionation methods: relevance to functional pools and to stabilization mechanisms. Soil Biology and Biochemistry.

[14] Lützow MV (2006). Stabilization of organic matter in temperate soils: mechanisms and their relevance under different soil conditions–a review. European Journal of Soil Science.

[15] Cui J (2014). Physical and chemical stabilization of soil organic carbon along a 500-year cultived soil chronosequence originating from estuarine wetlands: Temporal patterns and land use effects. *Agriculture, ecosystems &*. environment.

[16] Benbi DK, Kiranvir B, Sharma S (2015). Sensitivity of labile soil organic carbon pools to long-term fertilizer, straw and manure management in rice-wheat system. Pedosphere.

[17] Ozlu, E. & Kumar, S. Response of Soil Organic Carbon, pH, Electrical Conductivity, and Water Stable Aggregates to Long-Term Annual Manure and Inorganic Fertilizer. *Soil Science Society of America Journal* (2018).

[18] Šimon T (2008). The influence of long-term organic and mineral fertilization on soil organic matter. Soil Water Res.

[19] Franko U (1997). Modellierung des Umsatzes der organischen Bodensubstanz. Archives of Agronomy and Soil Science.

[20] Van Antwerpen. In *Proc S Afr Sug Technol Ass*. 179.

[21] Weil RR, Islam KR, Stine MA, Gruver JB, Samson-Liebig SE (2003). Estimating active carbon for soil quality assessment: A simplified method for laboratory and field use. American Journal of Alternative Agriculture.

[22] Fraser D, Doran J, Sahs W, Lesoing G (1988). Soil microbial populations and activities under conventional and organic management. Journal of Environmental Quality.

[23] Fauci MF, Dick R (1994). Soil microbial dynamics: short-and long-term effects of inorganic and organic nitrogen. Soil Science Society of America Journal.

[24] Vestal JR, White DC (1989). Lipid analysis in microbial ecology. Bioscience.

[25] Jindo K (2012). Biochar influences the microbial community structure during manure composting with agricultural wastes. Science of the Total Environment.

[26] Peacock AG (2001). Soil microbial community responses to dairy manure or ammonium nitrate applications. Soil Biology and Biochemistry.

[27] Davidson EA, Janssens IA (2006). Temperature sensitivity of soil carbon decomposition and feedbacks to climate change. Nature.

[28] Powlson D, Brookes P, Whitmore A, Goulding K, Hopkins D (2011). Soil organic matters. European Journal of Soil Science.

[29] Acosta-Martínez V, Upchurch D, Schubert A, Porter D, Wheeler T (2004). Early impacts of cotton and peanut cropping systems on selected soil chemical, physical, microbiological and biochemical properties. Biology and fertility of soils.

[30] Dodor DE, Ali Tabatabai M (2005). Glycosidases in soils as affected by cropping systems. Journal of Plant Nutrition and Soil Science.

[31] Moelans D, Cool P, Baeyens J, Vansant EF (2005). Using mesoporous silica materials to immobilise biocatalysis-enzymes. Catalysis Communications.

[32] García-Orenes F, Morugán-Coronado A, Zornoza R, Scow K (2013). Changes in soil microbial community structure influenced by agricultural management practices in a Mediterranean agro-ecosystem. PloS one.

[33] Goyal S, Chander K, Mundra M, Kapoor K (1999). Influence of inorganic fertilizers and organic amendments on soil organic matter and soil microbial properties under tropical conditions. Biology and Fertility of Soils.

[34] Kundu S, Bhattacharyya R, Prakash V, Ghosh B, Gupta H (2007). Carbon sequestration and relationship between carbon addition and storage under rainfed soybean–wheat rotation in a sandy loam soil of the Indian Himalayas. Soil and Tillage Research.

[35] McLauchlan KK, Hobbie SE (2004). Comparison of labile soil organic matter fractionation techniques. Soil Science Society of America Journal.

[36] Ghani A, Dexter M, Perrott K (2003). Hot-water extractable carbon in soils: a sensitive measurement for determining impacts of fertilisation, grazing and cultivation. Soil Biology and Biochemistry.

[37] Wilson HF, Xenopoulos MA (2009). Effects of agricultural land use on the composition of fluvial dissolved organic matter. Nature Geoscience.

[38] Giacometti C (2013). Chemical and microbiological soil quality indicators and their potential to differentiate fertilization regimes in temperate agroecosystems. Applied Soil Ecology.

[39] Si G (2017). Changes in soil microbial community composition and organic carbon fractions in an integrated rice–crayfish farming system in subtropical China. Scientific reports.

[40] Potthast K, Hamer U, Makeschin F (2012). Land-use change in a tropical mountain rainforest region of southern Ecuador affects soil microorganisms and nutrient cycling. Biogeochemistry.

[41] Zhang Q-C (2012). Chemical fertilizer and organic manure inputs in soil exhibit a vice versa pattern of microbial community structure. Applied Soil Ecology.

[42] De Vries FT, Hoffland E, van Eekeren N, Brussaard L, Bloem J (2006). Fungal/bacterial ratios in grasslands with contrasting nitrogen management. Soil Biology and Biochemistry.

[43] Zhang Q-c, Wang G-h, Yao H-y (2007). Phospholipid fatty acid patterns of microbial communities in paddy soil under different fertilizer treatments. Journal of Environmental Sciences(China).

[44] Rasmussen K (1999). Impact of ploughless soil tillage on yield and soil quality: a Scandinavian review. Soil and Tillage Research.

[45] Zhong W (2010). The effects of mineral fertilizer and organic manure on soil microbial community and diversity. Plant Soil.

[46] Frostegård Å, Petersen SO, Bååth E, Nielsen TH (1997). Dynamics of a microbial community associated with manure hot spots as revealed by phospholipid fatty acid analyses. Applied and environmental microbiology.

[47] Hackl E, Pfeffer M, Donat C, Bachmann G, Zechmeister-Boltenstern S (2005). Composition of the microbial communities in the mineral soil under different types of natural forest. Soil Biology and Biochemistry.

[48] Liu Y, Lu H, Yang S, Wang Y (2016). Impacts of biochar addition on rice yield and soil properties in a cold waterlogged paddy for two crop seasons. Field crops research.

[49] Ozlu E, Kumar S (2018). Response of surface GHG fluxes to long-term manure and inorganic fertilizer application in corn and soybean rotation. Science of the Total Environment.

[50] Kandeler E, Gerber H (1988). Short-term assay of soil urease activity using colorimetric determination of ammonium. Biology and fertility of Soils.

[51] Dick, R. P. *Methods of soil enzymology*. (Soil Science Society of America Madison, WI, 2011).

[52] Silveira M, Comerford N, Reddy K, Cooper W, El-Rifai H (2008). Characterization of soil organic carbon pools by acid hydrolysis. Geoderma.

[53] Frostegård Å, Tunlid A, Bååth E (1993). Phospholipid fatty acid composition, biomass, and activity of microbial communities from two soil types experimentally exposed to different heavy metals. Applied and Environmental Microbiology.

[54] SAS. SAS Data Integration Studio 4.9: User’s Guide. SAS Institute (2014).

[55] Xue PP, Carrillo Y, Pino V, Minasny B, McBratney AB (2018). Soil Properties Drive Microbial Community Structure in a Large Scale Transect in South Eastern Australia. Scientific reports.

[56] Zhao C (2019). Dynamics of soil microbial communities following vegetation succession in a karst mountain ecosystem, Southwest China. Scientific reports.

[57] Dormaar, J. F., Lindwall, C. W. & Kozub, G. C. Effectiveness of manure and commercial fertilizer in restoring productivity of an arti®cially eroded dark brown chernozemic soil under dryland conditions. *Canadian Journal of Soil Science* **68**, 669 ± 679 (1988).

[58] Peacock AD (2001). Soil microbial community responses to dairy manure or ammonium nitrate applications. Soil Biol Biochem.

[59] Larkin RP, Honeycutt CW, Griffin TS (2006). Effect of swine and dairy manure amendments on microbial communities in three soils as influenced by environmental conditions. Biology and Fertility of Soils.

