# Supplementary material for: Soil health indicators impacted by long-term cattle manure and inorganic fertilizer application in a corn-soybean rotation of South Dakota
Source: Sci Rep. 2019 Aug 13;9:11776. doi: 10.1038/s41598-019-48207-z (PMC6692313; doi:10.1038/s41598-019-48207-z)
Supplement: Supplementary file 1 — Supplementary Info [file 41598_2019_48207_MOESM1_ESM.docx]

**Soil health indicators impacted by long-term cattle manure and inorganic fertilizer of corn-soybean rotation in South Dakota**

**Ekrem Ozlu^1^** ([eozlu@wisc.edu](mailto:eozlu@wisc.edu))

*Correspondence and requests for materials should be addressed to Ekrem Ozlu.

^1^Department of Soil Science, University of Wisconsin-Madison, Madison, WI 53706, USA.

**Saroop S. Sandhu^3^ (sss67@ufl.edu)**

^3^Department of Soil and Water Sciences, University of Florida, Gainesville, Florida, USA.

**Sandeep Kumar^2^ (sandeep.kumar@sdstate.edu)**

^2^Department of Agronomy, Horticulture and Plant Sciences, South Dakota State University, Brookings, SD 57007, USA,

**Francisco Arriaga^1^ (farriaga@wisc.edu)**

^1^Department of Soil Science, University of Wisconsin-Madison, Madison, WI 53706, USA.

| **Analysis** | **Units** | **Dairy Manure^2^** |
| --- | --- | --- |
| Total N | ------kg/ton------ | 5.93 |
| NH4-N |  | 2.60 |
| Organ. N |  | 3.33 |
| Avail N |  | 3.14 |
| P2O5 |  | 2.45 |
| K2O |  | 4.19 |
| Moisture | % | 72.08 |

**S1. Average Manure Analysis Results at Brookings (Dairy Manure; 2008-2016).** ^1^ Manure was collected and analyzed. ^2^Dairy manure collected in the spring to apply in the Brookings felt research farm.

| **TRT** | **Manure Applied** | **--------Manure--------** | | | **----------------Fertilizer----------------** | | | | |
| --- | --- | --- | --- | --- | --- | --- | --- | --- | --- |
|  |  | **N_2_** | **P_2_O_5_** | **K_2_O** | **N_2_** | **P_2_O_5_** | **K_2_O** | **Zinc** | **Sulfur** |
|  | ----------------------------- Brookings kg/ha ------------------------------------ | | | | | | | | |
| LM | 8.77 | 149.70 | 55.79 | 69.49 | 0.00 | 0.00 | 0.00 | 0.00 | 0.00 |
| MM | 20.54 | 225.91 | 104.36 | 173.98 | 0.00 | 0.00 | 0.00 | 0.00 | 0.00 |
| HM | 37.55 | 222.72 | 193.28 | 315.71 | 0.00 | 0.00 | 0.00 | 0.00 | 0.00 |
| MF | 0.00 | 0.00 | 0.00 | 0.00 | 40.72 | 18.56 | 22.42 | 0.00 | 0.00 |
| HF | 0.00 | 0.00 | 0.00 | 0.00 | 124.54 | 69.74 | 82.20 | 8.10 | 28.02 |
| CK | 0.00 | 0.00 | 0.00 | 0.00 | 0.00 | 0.00 | 0.00 | 0.00 | 0.00 |

**S2. The average nutrient contents of each treatment applied at Brookings (Dairy Manure; 2008-2016).** ^†^Mean values followed by different lower letters between each treatment within each sampling time represent significant differences due to manure and inorganic fertilizer application at *P*<0.05. ^††^LM, low manure rate based on recommended phosphorus rate; MM, medium manure rate based on recommended nitrogen rate; HM, high manure rate based on double of the recommended nitrogen rate; MF, recommended fertilizer; HF, high fertilizer; and CK, control with no manure application; TRT, treatments. Manure and fertilizer treatments (lbs. acre^-1^*0.00112=kg ha^-1^, acre*2.4711=ha, tons/ha*0.907185=mega gram (Mg) ha^-1^).

| **Treatments** | **Urease** | | |  | **β-Glucosidase** | | |
| --- | --- | --- | --- | --- | --- | --- | --- |
|  | ***(****µg NH_4_-N g^-1^ soil h^-1^****)*** | | |  | ***(****µmol p-nitrophenol g^-1^soil h^-1^****)*** | | |
|  | **P** | **1MAP** | **H** |  | **P** | **1MAP** | **H** |
| LM^††^ | 30.7^a†^ | 19.6^a^ | 21.6^bac^ |  | 22.2^bc^ | 24.2^ba^ | 25.4^bac^ |
| MM | 28.5^ba^ | 20.1^ba^ | 24.7^ba^ |  | 20.9^c^ | 26.3^a^ | 26.4^ba^ |
| HM | 27.9^ba^ | 19.7^a^ | 27.3^a^ |  | 24.8^a^ | 24.2^ba^ | 27.8^a^ |
| MF | 22.5^c^ | 17.8^b^ | 21.0^bc^ |  | 23.6^ba^ | 23.0^b^ | 22.2^d^ |
| HF | 23.3^bc^ | 18.5^b^ | 19.8^bc^ |  | 21.7^c^ | 23.6^b^ | 24.0^bc^ |
| CK | 22.5^c^ | 19.5^a^ | 17.1^c^ |  | 21.3^c^ | 19.7^c^ | 19.8^d^ |
| Analysis of Variance (*P>F*) | | | | | | | |
| *Treatment* | 0.020 | 0.0002 | 0.040 |  | 0.001 | 0.0004 | 0.001 |
| M vs. F | 0.0001 | 0.0004 | 0.056 |  | 0.900 | 0.030 | 0.010 |

**S3. Soil enzyme activities as impacted by long-term manure and fertilizer treatments for 0-7.5 cm depth at soybean planting (P), one month after soybean planting (1MAP) and at soybean harvest (H).** ^†^Mean values followed by different lower letters between each treatment within each sampling time represent significant differences due to manure and inorganic fertilizer application at *P*<0.05. ^††^LM, low manure rate based on recommended phosphorus rate; MM, medium manure rate based on recommended nitrogen rate; HM, high manure rate based on double of the recommended nitrogen rate; MF, recommended fertilizer; HF, high fertilizer; and CK, control with no manure application.

| **Treatments** | **CWECN** | | |  | **HWECN** | | |  | **AECN** | | |
| --- | --- | --- | --- | --- | --- | --- | --- | --- | --- | --- | --- |
|  | **P** | **1MaP** | **H** |  | **P** | **1MaP** | **H** |  | **P** | **1MaP** | **H** |
| LM^††^ | 3.60^ns^ | 4.66^a^ | 4.41^ns^ |  | 6.03^b^ | 6.13^ns^ | 6.25^ns^ |  | 5.20^ns^ | 4.92^ba^ | 5.77^ns^ |
| MM | 3.46 | 5.37^a^ | 3.57 |  | 5.35^b^ | 5.78 | 6.08 |  | 5.29 | 5.14^ba^ | 5.43 |
| HM | 4.40 | 2.93^ba^ | 4.00 |  | 4.95^a^ | 7.12 | 5.54 |  | 4.92 | 5.50^bc^ | 5.18 |
| MF | 2.94 | 2.56^bc^ | 4.42 |  | 6.96^a^ | 9.53 | 6.93 |  | 4.79 | 4.74^bc^ | 6.06 |
| HF | 3.00 | 3.05^c^ | 7.07 |  | 7.00^b^ | 6.83 | 7.80 |  | 5.18 | 5.02^c^ | 5.77 |
| CK | 3.09 | 2.47^a^ | 5.16 |  | 6.97^b^ | 7.44 | 7.17 |  | 5.56 | 5.23^a^ | 5.19 |
|  | *Analysis of Variance (P>F)* | | | | | | | |  |  |  |
| \| Treatments \| \| --- \| | 0.080 | 0.001 | 0.135 |  | 0.003 | 0.121 | 0.602 |  | 0.320 | 0.024 | 0.459 |
| M vs. F | 0.003 | 0.003 | 0.513 |  | 0.0002 | 0.074 | 0.346 |  | 0.015 | 0.018 | 0.252 |

**S4. Soil carbon and nitrogen ratio in different fractions (CWECN, carbon and nitrogen ratio in cold water exchangeable fraction; HWECN, carbon and nitrogen ratio in hot water exchangeable fraction; AECN, carbon and nitrogen ratio in acid exchangeable fraction) as impacted by long-term manure and fertilizer treatments for 0-7.5 cm depth at soybean planting (P), one month after soybean planting (1MAP) and at soybean harvest (H).** ns, no significant.
